# Androgen deprivation therapy prevents bladder cancer recurrence

**DOI:** 10.18632/oncotarget.2851

**Published:** 2014-12-24

**Authors:** Koji Izumi, Masataka Taguri, Hiroshi Miyamoto, Yoshinori Hara, Takeshi Kishida, Kimio Chiba, Tetsuo Murai, Kotaro Hirai, Kotaro Suzuki, Kiyoshi Fujinami, Teiichiro Ueki, Koichi Udagawa, Kazuo Kitami, Masatoshi Moriyama, Yasuhide Miyoshi, Futoshi Tsuchiya, Ichiro Ikeda, Kazuki Kobayashi, Maho Sato, Satoshi Morita, Kazumi Noguchi, Hiroji Uemura

**Affiliations:** ^1^ Department of Urology, Yokohama City University Graduate School of Medicine, Yokohama, Japan; ^2^ Department of Biostatistics and Epidemiology, Yokohama City University Medical Center, Yokohama, Japan; ^3^ Departments of Pathology and Urology, Johns Hopkins University School of Medicine, Baltimore, MD, USA; ^4^ Department of Urology, Odawara Municipal Hospital, Odawara, Japan; ^5^ Department of Urology, Kanagawa Cancer Center, Yokohama, Japan; ^6^ Department of Urology, Kawasaki Municipal Ida Hospital, Kawasaki, Japan; ^7^ Department of Urology, International Goodwill Hospital, Yokohama, Japan; ^8^ Department of Urology, Sagamihara National Hospital, Sagamihara, Japan; ^9^ Department of Urology, Saiseikai Yokohamashi Nanbu Hospital, Yokohama, Japan; ^10^ Department of Urology, Chigasaki Municipal Hospital, Chigasaki, Japan; ^11^ Department of Urology, Japanese Red Cross Hadano Hospital, Hadano, Japan; ^12^ Department of Urology, Hiratsuka Kyousai Hospital, Hiratsuka, Japan; ^13^ Department of Urology, Fujisawa City Hospital, Fujisawa, Japan; ^14^ Department of Urology, Yokohama Municipal Citizen's Hospital, Yokohama, Japan; ^15^ Department of Urology, Yokohama City University Medical Center, Yokohama, Japan; ^16^ Department of Urology, Yokohama City Minato Red Cross Hospital, Yokohama, Japan; ^17^ Department of Urology, Yokohama Minami Kyousai Hospital, Yokohama, Japan; ^18^ Department of Urology, Yokosuka Kyousai Hospital, Yokosuka, Japan; ^19^ Department of Biomedical Statistics and Bioinformatics, Kyoto University Graduate School of Medicine, Kyoto, Japan

**Keywords:** Bladder cancer, recurrence, androgen, androgen deprivation therapy

## Abstract

Although accumulating preclinical evidence indicates the involvement of androgen receptor signals in bladder cancer (BC) development, its clinical relevance remains unclear. We aimed to evaluate the predictive role of androgen deprivation therapy (ADT) in BC recurrence in prostate cancer (PC) patients.

We retrospectively reviewed 20,328 patients with PC diagnosed during 1991–2013 and identified 239 (1.2%) men having primary BC. After excluding ineligible patients, 162 patients made up a final cohort.

With a median follow-up of 62 months, 38 (50%) of 76 control patients without ADT experienced BC recurrence, while 19 (22%) of 86 did in ADT group. Thus, patients having received ADT for their PC showed a significantly lower risk of BC recurrence (5-year actuarial recurrence-free survival: 76% *v* 40%; *P* < 0.001) and also had a significantly smaller number of recurrence episodes (5-year cumulative recurrence: 0.44 *v* 1.54; *P* < 0.001), compared to the control patients. A multivariable analysis revealed ADT as an independent prognosticator (hazard ratio, 0.29; 95% confidence interval, 0.17–0.49) for BC recurrence.

This is the first clinical study showing that ADT significantly reduces the risk of BC recurrence.

## INTRODUCTION

Urinary bladder cancer (BC) is the fourth and seventh most common malignancy in the US [1] and worldwide [2], respectively, in men. Most BCs are initially non-muscle-invasive and can be treated by transurethral resection (TUR). However, the recurrence rate of BC is quite high (50%–70%) and progression to invasive state takes place occasionally [3]. Although bladder instillation therapy using anthracyclines or Bacillus Calmette-Guérin (BCG) is reported to be effective, the risk of recurrence even after the instillation treatment remains as high as 36%–51% [3]. In addition, due to the lifelong necessity to monitor the recurrence of BC, the medical cost per BC patient from diagnosis to death is the highest among all cancer types [4].

One intriguing feature in BC is the three- to four-fold higher incidence in men than in women [1, 2]. Although excessive exposure to industrial chemicals and cigarette smoke may have contributed to this male predilection, men remain at a substantially higher risk of BC than women even after controlling for these carcinogenic factors [5, 6]. These findings prompted us to fathom the mechanism underlying such male predilection. In fact, we and others have found molecular evidence indicating, in animal models, that androgen receptor (AR) signals are involved in BC development [7–9]. It is therefore suggested that androgen-mediated activation of AR signaling would promote bladder tumorigenesis and cancer progression. There are basically two relevant states which may clearly explain the male predilection of BC. First, non-neoplastic urothelial and BC tissues in men express higher levels of AR and/or molecules related to its downstream pathway than those in women. Second, more simply, higher levels of circulating androgens in men would stimulate and maintain higher activation state of AR signaling in urothelial/carcinoma cells. Examinations on the first hypothesis failed to demonstrate it to be the case [9–13]. Clinical studies have neither been successful to reveal the relationship between clinical course of patients or pathological state of BCs and AR level in BCs. On the other hand, the second hypothesis has not been examined so far in a well-controlled manner and remains to be an open question.

Prostate cancer (PC) is one of the most prevalent malignancies for men in developed countries [2]. As for its treatment, androgen deprivation therapy (ADT) stands as a critical treatment option [14]. It is used primarily for advanced PC in Europe and North America, while more widely used in Japan, even for some localized PC patients [15]. Interestingly, patients with PC are known to have a higher risk of coincidental BC, reported to be 1.2–3.4% of PC patients, than otherwise healthy men, probably due to shared molecular carcinogenic process between these two cancers [16].

Upon reviewing the background information, we have inclined to consider that circulating androgens would be responsible for AR-mediated gender difference in bladder tumorigenesis and cancer progression. If that is the case, we reasoned that ADT could influence the development of BC in PC patients. To test this hypothesis, we undertook a multicenter retrospective cohort study involving 20, 328 PC patients, including those having received ADT, and found surprisingly that ADT significantly reduced the risk of BC recurrence.

## RESULTS

### Patient characteristics

Table 1 shows the patient characteristics of the study cohort. The median follow-up for the entire cohort was 62 months (interquartile range, 19 to 95 months). Of the 162 patients, 86 were treated with ADT, and the remaining 76 did not receive ADT for their PC. The age of the patients was significantly higher in the ADT group than in the control group without ADT (*P* = 0.028). Among the reported clinicopathological factors of BC recurrence, i.e., tumor grade, stage, size, multiplicity and concomitant carcinoma in situ (CIS), the number of grade 2 tumors was significantly higher in the ADT group (*P* = 0.032). There was no significant difference in prophylactic bladder instillation therapy between the two groups. Regarding the features of PC, the prostate-specific antigen (PSA) level (*P* = 0.016) and tumor T stage (*P* = 0.047) were significantly higher, and brachytherapy was significantly less likely to have been performed (*P* < 0.001) in the ADT group compared to the control group.

**Table 1:** Baseline Characteristics of the Patients with and without Androgen Deprivation Therapy (ADT)

	Characteristics	Control, *n* (%)	ADT, *n* (%)	*P*
	No. of patients	76 (46.9)	86 (53.1)	
	Age^a^, y	71.5 (54–92)	74.0 (59–88)	0.028
BC	Tumor grade			0.032
	1	26 (35.6)	16 (20.5)	
	2	27 (37.0)	45 (57.7)	
	3	20 (27.4)	17 (21.8)	
	Pathological T stage			0.118
	Ta	54 (75.0)	51 (62.2)	
	≥T1	18 (25.0)	31 (37.8)	
	Tumor size			1.000
	<3 cm	52 (81.3)	59 (81.9)	
	≥3 cm	12 (18.8)	13 (18.1)	
	Tumor number			0.230
	Single	33 (52.4)	30 (41.7)	
	Multiple	30 (47.6)	42 (58.3)	
	Concomitant CIS			0.241
	No	60 (89.6)	64 (82.1)	
	Yes	7 (10.4)	14 (17.9)	
	Instillation			0.087
	No	41 (55.4)	40 (48.2)	
	Anthracyclines	21 (28.4)	17 (20.5)	
	BCG	12 (16.2)	26 (31.3)	
PC	PSA^b^, ng/ml	7.9 (5.2–13.0)	9.7 (6.8–17.9)	0.016
	Gleason score			0.051
	≤6	30 (43.5)	21 (25.0)	
	7	22 (31.9)	33 (39.3)	
	≥8	17 (24.6)	30 (35.7)	
	Clinical T stage			0.047
	T1	47 (64.4)	42 (50.6)	
	T2	23 (31.5)	26 (31.3)	
	T3	3 (4.1)	14 (16.9)	
	T4	0 (0.0)	1 (1.2)	
	Clinical N stage			0.500
	N0	72 (100.0)	82 (97.6)	
	N1	0 (0.0)	2 (2.4)	
	Clinical M stage			0.123
	M0	75 (100.0)	81 (95.3)	
	M1	0 (0.0)	4 (4.7)	
	External beam radiotherapy			1.000
	No	61 (87.1)	67 (87.0)	
	Yes	9 (12.9)	10 (13.0)	
	Brachytherapy			<0.001
	No	47 (70.1)	71 (93.4)	
	Yes	20 (29.9)	5 (6.6)	
	Radical prostatectomy			0.227
	No	59 (88.1)	72 (94.7)	
	Yes	8 (11.9)	4 (5.3)	

a Numbers are median (range).

b Numbers are median (interquartile range).

Abbreviations: ADT, androgen deprivation therapy; BC, bladder cancer; PC, prostate cancer; CIS, carcinoma in situ; BCG, Bacillus Calmette–Guérin; PSA, prostate-specific antigen.

### ADT and recurrence-free survival (RFS)

We performed a Kaplan-Meier analysis coupled with a log-rank test to assess possible associations between ADT and BC recurrence. Overall, 19 (22%) of the 86 patients with ADT versus 38 (50%) of the 76 patients without ADT developed BC recurrence (*P* < 0.001). The 5-year actuarial RFS rates for the ADT and control patients were 76% and 40%, respectively. The ADT patients thus showed significantly improved RFS compared to the control patients without ADT (*P* < 0.001; Fig. 2). To further explore the time-response relationship, we subdivided the patients into three groups according to ADT proportion that was defined as (treatment period with ADT)/(observed period for the first recurrence). The 5-year actuarial RFS rates based on ADT proportion (0%, ≤50%, and >50%) were 40%, 63%, and 82%, respectively, which were significantly different (*P* < 0.001; Supplementary Fig. S1). These results indicate that ADT prevents BC recurrence in a time-dependent manner.

**Figure 1:**
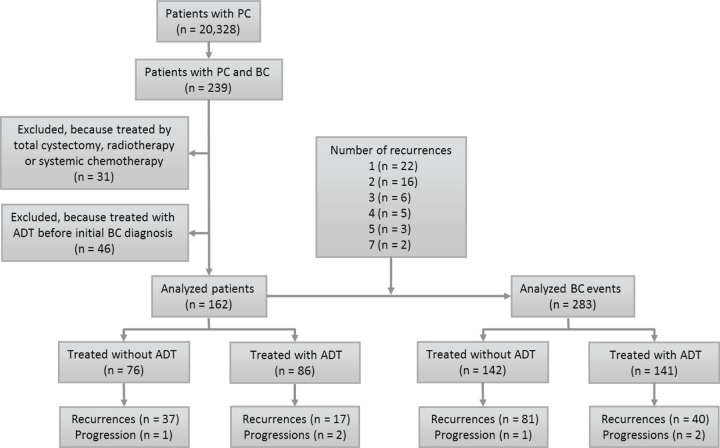
Flow of participants through study

**Figure 2:**
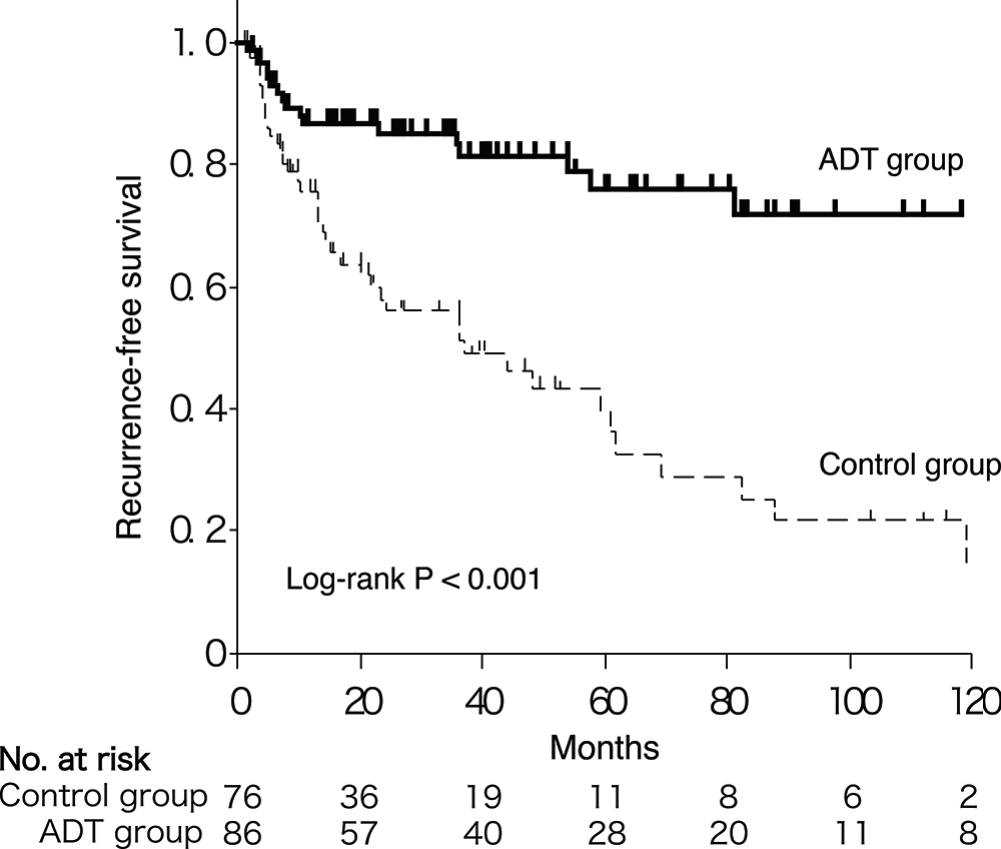
Kaplan-Meier curves for RFS in BC patients with versus without ADT

### ADT and the number of recurrence episodes

Because BC patients often have multiple episodes of recurrence, we additionally analyzed the cumulative recurrence between the ADT and control groups. Among the 162 patients, 22 patients developed one recurrence, and 32 patients had more than one recurrence: two recurrences (*n* = 16), three (*n* = 6), four (*n* = 5), five (*n* = 3), and seven (*n* = 2). Consequently, these patients had a total of 283 BC events (initial BCs and recurrent BCs), including 141 in the ADT group and 142 in the control group (Fig. 1).

**Figure 3:**
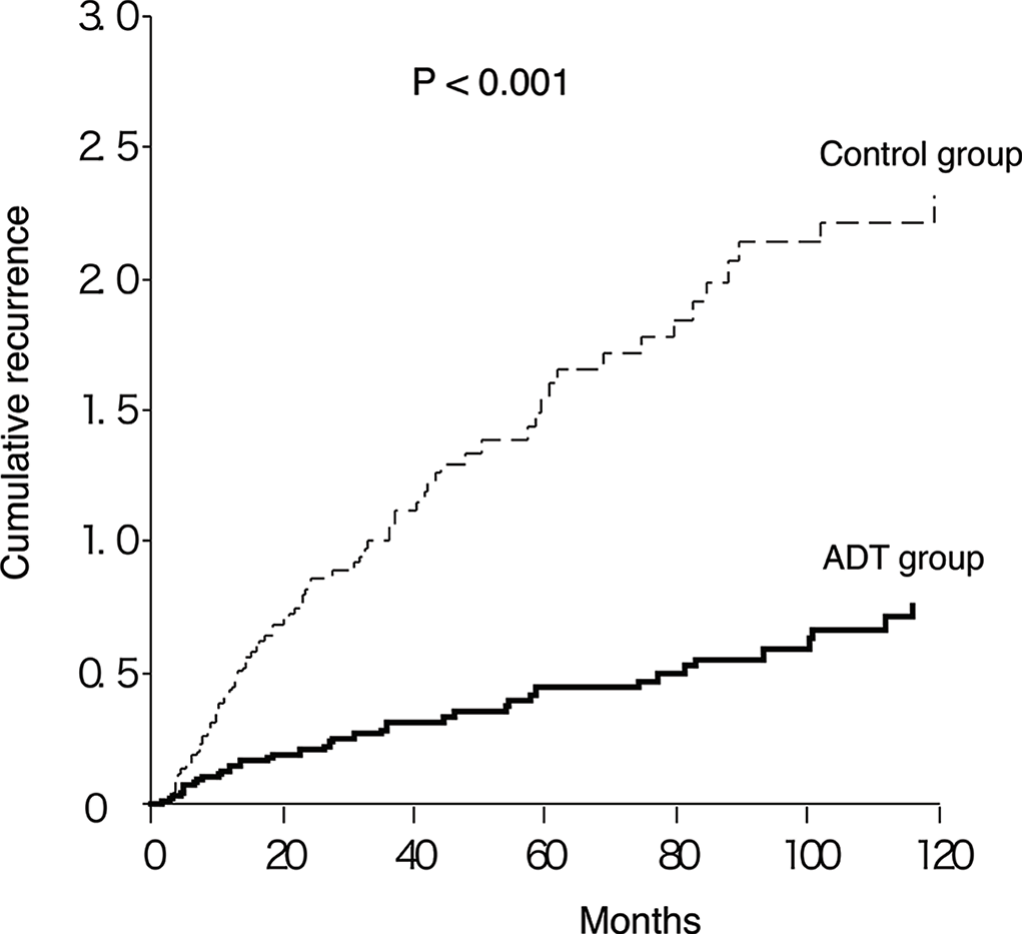
Cumulative recurrence of BC in patients with versus without ADT

There were no statistically significant differences in the histopathologic features of BC between the ADT and control groups (Table 2). We then compared the cumulative recurrences of BC between the two groups. The number of 5-year cumulative recurrence episodes was significantly smaller in the ADT group than in the control group (0.44 *v* 1.54; *P* < 0.001; Fig. 3). A Cox regression analysis demonstrated a lower risk of recurrence in the ADT group (hazard ratio (HR), 0.29; 95% confidence interval (CI), 0.19–0.45; Table 3). Older age was also an independent predictor for recurrence (HR, 1.06; 95% CI, 1.03–1.09; Table 3).

**Table 2:** Clinicopathological Features of All BC Events with and without Androgen Deprivation Therapy (ADT)

Characteristics	Control, *n* (%)	ADT, *n* (%)	*P*
No. of events	142 (50.2)	141 (49.8)	
Age^a^, y	74.0 (54–93)	76.0 (59–90)	0.011
Tumor grade			1.000
1	44 (32.8)	30 (23.4)	
2	61 (45.5)	72 (56.3)	
3	29 (21.6)	26 (20.3)	
Pathological T stage			0.223
Ta	102 (75.6)	92 (68.1)	
≥T1	33 (24.4)	43 (31.9)	
Tumor size			0.442
<3 cm	106 (89.1)	101 (85.6)	
≥3 cm	13 (10.9)	17 (14.4)	
Tumor number			0.697
Single	55 (46.2)	52 (43.3)	
Multiple	64 (53.8)	68 (56.7)	
Concomitant CIS			0.861
No	108 (85.0)	110 (85.9)	
Yes	19 (15.0)	18 (14.1)	
Instillation			1.000
No	73 (53.3)	68 (50.0)	
Anthracyclines	44 (32.1)	32 (23.5)	
BCG	20 (14.6)	36 (26.5)	

a Numbers are median (range).

Abbreviations: ADT, androgen deprivation therapy; CIS, carcinoma in situ; BCG, Bacillus Calmette–Guérin.

**Table 3:** Univariable and Multivariable Analyses for Cumulative Recurrence

		Univariable analysis		Multivariable analysis	
	Variables	HR (95%CI)	*P*	HR (95%CI)	*P*
	ADT (yes vs. no)	0.29 (0.17–0.49)	<0.001	0.29 (0.19–0.45)	<0.001
	Age, continuous value	1.04 (1.01–1.07)	0.005	1.06 (1.03–1.09)	<0.001
BC	Tumor grade (2 vs. 1)	1.38 (0.80–2.38)	0.247	1.19 (0.72–1.96)	0.507
	Tumor grade (3 vs. 1)	1.10 (0.52–2.31)	0.805	0.66 (0.31–1.41)	0.282
	pT stage (≥T1 vs. ≤Ta)	0.82 (0.51–1.33)	0.422	1.16 (0.61–2.18)	0.656
	Tumor size (≥3 cm vs. <3 cm)	1.02 (0.51–2.06)	0.952	1.04 (0.54–2.03)	0.903
	Tumor number (Multiple vs. Single)	1.02 (0.65–1.60)	0.933	1.08 (0.72–1.63)	0.712
	CIS (yes vs. no)	0.91 (0.44–1.91)	0.811	1.44 (0.67–3.09)	0.350
	Anthracylines (yes vs. no)	0.87 (0.54–1.41)	0.580	0.69 (0.43–1.10)	0.117
	BCG (yes vs. no)	0.47 (0.26–0.84)	0.011	0.59 (0.30–1.18)	0.137
PC	Gleason Score (≥7 vs. ≤6)	0.83 (0.43–1.61)	0.582		
	Gleason Score (≥8 vs. ≤6)	1.09 (0.56–2.13)	0.793		
	Gleason Score (≥8 vs. ≤7)	1.21 (0.68–2.13)	0.521		
	PSA, continuous value	1.05 (0.60–1.84)	0.865		
	cT stage (≥T3 vs. ≤T2)	1.36 (0.66–2.81)	0.402		
	cN stage (N1 vs. N0)	1.29 (0.12–13.37)	0.832		
	cM stage (M1 vs. M0)	1.75 (0.34–8.98)	0.501		
	EBRT (yes vs. no)	1.09 (0.57–2.07)	0.791		
	Brachytherapy (yes vs. no)	0.74 (0.25–2.20)	0.590		
	RP (yes vs. no)	1.21 (0.56–2.65)	0.628		

Abbreviations: ADT, androgen deprivation therapy; BC, bladder cancer; PC, prostate cancer; CIS, carcinoma in situ; BCG, Bacillus Calmette–Guérin; PSA, prostate-specific antigen; EBRT, external beam radiotherapy; RP, radical prostatectomy.

### Landmark analysis

In an analysis of the effect of ADT on RFS, the length of the RFS would influence the chance of receiving ADT, thereby inducing a bias in favor of ADT. To minimize this bias, we conducted a landmark analysis with 12 months from the diagnosis as the landmark time point [17]. This analysis included patients who were at risk 12 months after diagnosis (the landmark time as determined a priori), and it compared the outcomes of those who had and had not received ADT in the prior 12 months. The patient backgrounds were not significantly different between the two groups, except for older age in the ADT group (Supplementary Table S1). Consistent with the results of all patients, the performance of ADT improved the RFS (Supplementary Fig. S2).

## DISCUSSION

Molecular evidence has indicated that AR signals promote bladder carcinogenesis as well as cancer progression [9]. With the use of a chemical carcinogen N-butyl-N-(4-hydroxybutyl) nitrosamine (BBN)-induced BC model, castrated male and intact female mice were shown to have a lower incidence of BC compared to intact male mice. In addition, AR knockout in the entire body [7] or urothelium only [8] resulted in failure of BBN to induce BC. An AR degradation enhancer, ASC-J9, inhibited tumorigenesis in BBN-treated wild-type male mice [8]. Based on these data, androgen-mediated AR signaling in urothelial cells has been suggested to be a therapeutic target in BC. In the present study, we showed, for the first time, clinical evidence implying that ADT prevents BC recurrence.

Incidence of BC in 20,328 PC patients in the current study was 1.2%, which is consistent with previous findings [16]. Since we set the recurrence of BC as a primary endpoint instead of the incidence of BC, we excluded patients who had already received ADT before initial BC diagnosis in order to circumvent a possible ADT effect on the characteristics of the initial BC. We were unable to assess the effect of ADT on BC progression because only three patients in our cohort developed muscle invasion. Of the 31 patients who had been treated by radical cystectomy, radiotherapy, or systemic chemotherapy, and were excluded from the analysis, eight developed metastasis or local invasion, which resulted in cancer-specific mortality. The effect of ADT on cancer-specific survival among these patients remains unclear because of the relatively small sample size and complicated background. To further investigate the effect of ADT on BC progression, prospective trials involving patients undergoing radical cystectomy are required, as Gakis et al. have suggested [18].

In our cohort, men who received ADT were older and had higher PSA/stage PC than those without ADT, probably because ADT was more likely used for older patients with advanced PC. The patients who underwent brachytherapy had lower Gleason scores and, as expected, were less likely treated with ADT. Although the reason why the ADT group had more grade 2 BCs than the control group is unclear, ADT prevents BC recurrence even if cases are separately analyzed in each grade. In addition, among all of these clinicopathologic factors, only patient age was an independent prognosticator for BC recurrence in our multivariable analysis. The result of the age as one of the independent prognosticators for RFS in this study is consistent with the epidemiological finding indicating that elderly men have a higher incidence of BC. It has been reported that subsequent BC incidences are not different between the PC patients who underwent radical prostatectomy and radiation therapy [19]. Local treatments for PC (radical prostatectomy, external beam radiation therapy, and brachytherapy) had no or little influence on BC recurrence (Table 3).

We found that ADT dramatically prevented the first BC recurrence (Fig. 2). In addition, the ADT proportion correlated with the RFS (Supplementary Fig. S1), suggesting that the preventive effect of ADT on BC recurrence was time-dependent. Moreover, the cumulative recurrence analysis indicated that BC recurrences were again strongly suppressed (HR, 0.29; 95% CI, 0.17–0.49; Table 3) as long as the patients received ADT. Our landmark analysis excluded the possible bias that patients with longer RFS might have more likely received ADT.

We also observed that BCG instillation therapy tended to be more frequent in the ADT group, which might have affected the favorable RFS. However, our multivariable analysis revealed ADT, but not BCG instillation therapy, as an independent prognostic factor for BC recurrence (Table 3). These unexpected results further confirmed the preventive role of ADT in BC recurrence. Since gonadotropin-releasing hormone agonists were used as ADT forms in more than 90% of our patients, it remained unclear whether anti-androgen alone sufficiently prevented BC recurrence. Assessments of AR expression and androgen levels are needed to determine whether the preventive effect of ADT on BC recurrence is mediated by androgen-mediated AR signals or other possible pathways, such as estrogen receptor signals [20, 21].

Accumulating evidence suggests the involvement of various molecules that modulate AR activity and/or are regulated by AR signals in BC development. These included epidermal growth factor receptor/ERBB2 [12, 22, 23], Wnt/β-catenin [24, 25], p53 [8], UDP-glucuronosyltransferase-1A [26, 27], and CD24 [28]. The results of the present study strongly encourage further investigations of these downstream pathways of AR signals with the goal of developing a new therapeutic approach (in addition to standard forms of ADT) for BC.

The recurrence rate in BC patients undergoing bladder instillation therapy using an anthracycline or BCG remains high [3]. New preventive approaches for BC recurrence are thus urgently required. According to our cumulative recurrence analysis, roughly five person-years of ADT prevents one BC recurrence (Fig. 3). Because ADT has been widely used for PC and is generally well-tolerated, the clinical application of ADT to BC patients is feasible. We are planning to conduct a randomized clinical trial to further assess the efficacy of ADT in BC recurrence.

The limitation of the present study is that it was a multicenter retrospective study design. In the present study, reported prognosticators, such as tumor grade, stage, size, multiplicity and concomitant CIS [29], as well as prophylactic instillation therapy with BCG or anthracyclines, were not significantly associated with BC recurrence, probably due to the relatively small sample size.

In conclusion, in men with double primary cancers of the bladder and prostate, ADT for PC resulted in a significant decrease in the risk of BC recurrence. These results strongly suggest the involvement of androgen-AR signals in BC recurrence, as well as the efficacy and feasibility of ADT for patients with BC.

## METHODS

### Patients

Institutional review board approval was obtained to conduct this study. We retrospectively identified a total of 239 patients with primary BC, from 20,328 men with PC, between January 1991 and May 2013 at Yokohama City University and 15 affiliated hospitals. All BCs were graded in accord with the 1973 WHO classification systems for urothelial neoplasms [30], at each institution. Thirty-one patients were excluded because they had undergone radical cystectomy (*n* = 27), radiotherapy (*n* = 2), chemotherapy (*n* = 1), or chemoradiotherapy (*n* = 1) as an initial treatment for BC. Additional 46 patients who had already been treated with ADT before the initial diagnosis of BC were also excluded. The remaining 162 patients formed the study cohort for analysis (Fig. 1).

In general, the patients were followed every three months with cystoscopy for two years from the time of the initial treatment of their BC and every six months thereafter. All BC recurrences were pathologically confirmed in TUR specimens. Three patients who progressed to muscle-invasive BC were determined as recurrence and censored. The outcome measures included RFS and cumulative recurrence of BC. The ADT group included patients treated with ADT for PC at any time from the diagnosis of BC to recurrence or censoring.

### Statistical analysis

For the continuous variables, median and IQRs were reported. The categorical variables were summarized as frequencies and percentages. We evaluated the differences in the distribution of variables, using the chi-square test for categorical variables and a two-sample *t*-test or the Wilcoxon rank sum test for continuous variables, as appropriate. RFS defined as the time from the diagnosis of BC to the confirmation of disease recurrence or censoring was estimated by the Kaplan-Meier method and compared using the log-rank test. To examine the relationship between the time and mean number of recurrences, we calculated the mean cumulative function using ADT as the time-dependent covariate [31]. We estimated HRs and corresponding 95% CIs for the time to first and subsequent recurrences, using Univariable and multivariable Andersen and Gill models [32]. All statistical tests were two-tailed, and *P*-values < 0.05 were considered significant. Statistical analyses were performed using SAS statistical software, version 9.2 (SAS Institute, Cary, NC).

## SUPPLEMENTARY MATERIAL FIGURES AND TABLE


