# Supplementary material for: Androgen deprivation therapy prevents bladder cancer recurrence
Source: Oncotarget. 2014 Dec 24;5(24):12665–74. doi: 10.18632/oncotarget.2851 (PMC4350350; doi:10.18632/oncotarget.2851)
Supplement: Supplementary file 1 [file oncotarget-05-12665-s001.pdf]

## SUPPLEMENTARY FIGURES AND TABLE

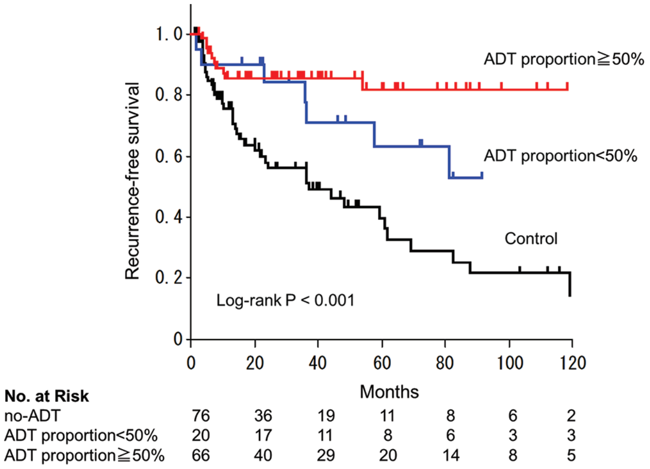


**Supplementary Figure S1: Kaplan-Meier curves for recurrence-free survival in bladder cancer patients according to androgen deprivation therapy (ADT) proportion.**

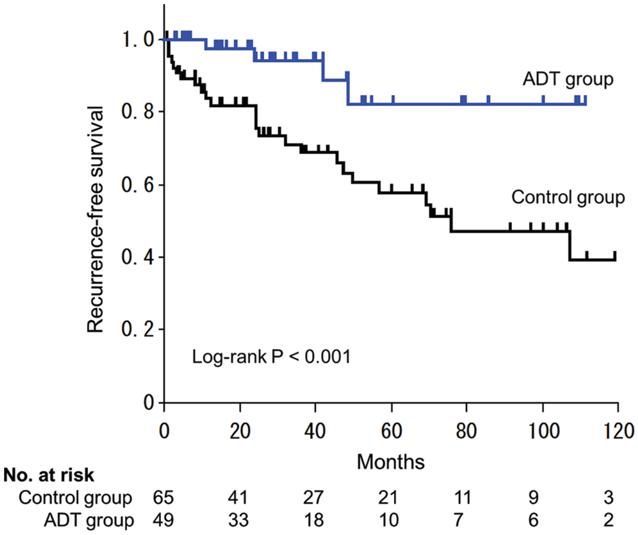


**Supplementary Figure S2: Kaplan-Meier curves for recurrence-free survival in bladder cancer patients with versus without ADT in landmark analysis.**

**Supplementary Table S1: Baseline Characteristics of the Patients with and without Androgen Deprivation Therapy (ADT) in Landmark Analysis**

| | Characteristics | Control, *n* (%) | ADT, *n* (%) | *P* |
|---|---|---|---|---|
| | No. of patients | 65 (57) | 49 (43) | |
| | Age[a], y | 72.0 (54–91) | 74.0 (64–92) | 0.016 |
| BC | Tumor grade | | | 0.071 |
| | 1 | 25 (39.7) | 8 (18.6) | |
| | 2 | 26 (41.3) | 25 (58.1) | |
| | 3 | 12 (19.0) | 10 (23.3) | |
| | Pathological T stage | | | 0.201 |
| | Ta | 47 (75.8) | 29 (63.0) | |
| | ≥T1 | 15 (24.2) | 17 (37.0) | |
| | Tumor size | | | 0.573 |
| | <3 cm | 44 (86.3) | 33 (80.5) | |
| | ≥3 cm | 7 (13.7) | 8 (19.5) | |
| | Tumor number | | | 0.291 |
| | Single | 27 (52.9) | 16 (40.0) | |
| | Multiple | 24 (47.1) | 24 (60.0) | |
| | Concomitant CIS | | | 0.572 |
| | No | 51 (87.9) | 36 (83.7) | |
| | Yes | 7 (12.1) | 7 (16.3) | |
| | Instillation | | | 0.675 |
| | No | 33 (52.4) | 20 (43.5) | |
| | Anthracyclines | 15 (23.8) | 13 (28.3) | |
| | BCG | 15 (23.8) | 13 (28.3) | |
| PC | PSA[b], ng/ml | 8.8 (6.4–14.0) | 8.3 (6.0–18.0) | 0.988 |
| | Gleason score | | | 0.056 |
| | ≤6 | 27 (42.2) | 9 (20.5) | |
| | 7 | 20 (31.3) | 19 (43.2) | |
| | ≥8 | 17 (26.6) | 16 (36.4) | |
| | Clinical T stage | | | 0.509 |
| | T1 | 36 (58.1) | 26 (53.1) | |
| | T2 | 21 (33.9) | 15 (30.6) | |
| | T3 | 5 (8.1) | 7 (14.3) | |
| | T4 | 0 (0.0) | 1 (2.0) | |
| | Clinical N stage | | | 1.000 |
| | N0 | 61 (98.4) | 49 (100.0) | |
| | N1 | 1 (1.6) | 0 (0.0) | |

(*Continued*)

| | Characteristics | Control, *n* (%) | ADT, *n* (%) | *P* |
|---|---|---|---|---|
| | Clinical M stage | | | 1.000 |
| | M0 | 63 (98.4) | 48 (98.0) | |
| | M1 | 1 (1.6) | 1 (2.0) | |
| | External beam radiotherapy | | | 0.789 |
| | No | 52 (83.9) | 38 (86.4) | |
| | Yes | 10 (16.1) | 6 (13.6) | |
| | Brachytherapy | | | 0.575 |
| | No | 49 (83.1) | 39 (88.6) | |
| | Yes | 10 (16.9) | 5 (11.4) | |
| | Radical prostatectomy | | | 1.000 |
| | No | 56 (91.8) | 39 (90.7) | |
| | Yes | 5 (8.2) | 4 (9.3) | |

[a]Numbers are median (range). [b]Numbers are median (interquartile range).
Abbreviations: ADT,androgen deprivation therapy; BC,bladder cancer; PC,prostate cancer; CIS,carcinoma in situ; BCG,Bacillus Calmette–Guérin; PSA,prostate-specific antigen.
